# ‘The GP’s offices are far away and the gain would be great.’—A qualitative study on nursing home staff’s perspectives on digital solutions for communication issues with general practitioners

**DOI:** 10.1080/02813432.2026.2638515

**Published:** 2026-04-06

**Authors:** Kathleen Denny, Jana Gundlack, Jonathan Bay, Eric Kroeber, Susanne Unverzagt, Alexander Bauer, Thomas Frese, Amand Führer

**Affiliations:** a Institute of General Practice & Family Medicine, Interdisciplinary Center of Health Sciences, Medical Faculty of Martin Luther University Halle-Wittenberg, Halle (Saale), Germany; b Institute for Medical Epidemiology, Biometrics and Informatics, Interdisciplinary Center of Health Sciences, Medical Faculty of Martin Luther University Halle-Wittenberg, Halle (Saale), Germany

**Keywords:** Nursing homes, general practitioners, interprofessional collaboration, digital options, paradox theory

## Abstract

**Background:**

An aging society and a growing shortage of healthcare professionals pose increasing challenges for nursing homes in Germany. Enhanced collaboration between nursing home staff and General Practitioners is essential to ensure quality care. Understanding nurses’ perspectives is crucial to identifying current issues and suitable solutions.

**Aim:**

To explore nursing home staff’s perspectives on communication and current channels with General Practitioners and evaluate specifically their attitudes towards digital communication options.

**Methods:**

This qualitative study involved semi-structured interviews with 13 nursing home employees from seven German nursing homes, conducted between January 2020 and April 2022. Data were transcribed verbatim and analysed thematically. Reporting adhered to COREQ guidelines. We used paradox theory from management studies to interpret communication tensions and solution strategies.

**Results:**

We identified three key themes: (1) Interprofessional communication problems between nurses and general practitioners, including limited direct contact, technical barriers, and time-related inefficiencies; (2) Perceived ability of digital options to reduce communication problems; and (3) Suggestions to improve communication, with a focus on digital options. Participants expressed a strong openness to digital communication tools, highlighting their potential to alleviate current tensions. Digital tools were viewed as opportunities to streamline communication, enhance efficiency, and ultimately improve collaboration and patient care.

**Conclusions:**

This study provides insights into tensions in healthcare collaboration in nursing homes and possible solution strategies. Participants see digital tools as promising pathways to resolve current inefficiencies and support better interprofessional collaboration. These insights are essential for guiding the development of practical, sustainable solutions to improve care delivery in nursing homes.

## 1. Introduction

### 1.1. New burdens in elderly care

Demographic shifts globally, and particularly in Germany, have increased the proportion of seniors (=aged ≥ 65), and intensify demands on healthcare systems [1]. In Germany, demographic change is most prominent in Saxony-Anhalt, with the highest proportion of seniors in the total population (27.8%) and one of the lowest densities of general practitioners (GPs)* [2–4]. In 2023, almost 800,000 residents lived in 16,500 nursing homes in Germany [5]. Simultaneously, the demand for nursing home staff is projected to increase by 39% from 2024 to 2049 [6]. German health care expenditures are high; yet, population health metrics are below the European average [7]. Combined with demographic change, a rising prevalence of frailty and increasing shortages of health care professionals, these facts place nursing homes in Germany at the forefront of health system challenges to come [8]. Addressing these challenges necessitates enhanced interprofessional collaboration, particularly between nursing homes and GPs.

### 1.2. Nursing homes in Germany

Nursing homes in Germany are defined in the German Social Code Book as institutions whose residents are fully inpatient and under permanent care by a registered nurse [6]. Nursing homes in Germany are operated by either the municipality, welfare organisations (e.g. church-based institutions), or private organisations [9]. Since 1996, health care in nursing homes is provided by the statutory long-term care insurance as an independent part of social insurance*. The-long-term care insurance in Germany does not cover all care costs, hence patients and relatives must also share the costs [10]. In a formalized process care needs are assessed and classified into different levels of care [10]. In contrast, in Sweden, care needs assessment is less formalized and carried out by care managers [11]. *GPs predominantly provide medical care for nursing home residents, who are free to choose their GP, and whilst the visit frequency varies between GPs, on average nursing homes receive a GP visit about once a week [12]. As shown in Table 1 below, nursing homes often have several collaborating GPs, so during a visit, a GP would normally only see their own patients. Physicians and nursing homes use a wide array of medical record systems. Thus, the government aims to standardise and digitalise medical records through electronic health records, but currently, success remains limited, mainly due to data security concerns [13].

**Table 1.** Sociodemographic characteristics of nursing home staff.

Code	Nursing home	Gender	Age	Education	Experience (years)	Current position	Collaborating GPs
1CB	A	Female	38	Geriatric Nurse	14	Head of Resident Care Unit & Instructor	4
2CB	A	Male	46	Nurse	5	Nursing Home Director	4
3PH	B	Female	46	Geriatric Nurse & Clerk	25	Head of Nursing (2 Homes)	>7
4ED	C	Female	37	Geriatric Nurse	20	Head of Resident Care Unit	10–15
5BH	B	Male	50	Administrator & MSc (Patient Management)	4	Nursing Home Director	<5
6DB	D	Female	46	Geriatric Nurse	28	Head of Resident Care Unit	4
7WH	E	Male	49	Geriatric Nurse	15	Head of Resident Care Unit	15
8WH	E	Female	53	Nurse	34	Head of Nursing	10–12
9XH	E	Female	56	Geriatric Nurse	14	Head of Nursing	10–12
10RR	F	Female	54	Pediatric Nurse	16	Head of Resident Care Unit	5
11AJ	G	Male	50	Geriatric Nurse	30	Deputy Head of Resident Care & Instructor	10–15
12BJ	G	Female	43	Nurse	10	Deputy Head of Resident Care Unit	15–20
13CJ	G	Male	30	Geriatric Nurse	8	Geriatric Nurse	10–15

Note: *For better readability, we will refer to all participating nursing home employees as nurses and use GP synonymously to Family Doctor. We will use nursing home staff and nurses interchangeably, with the latter highlighting the human dimension.

### 1.3. Interprofessional collaboration

The collaboration between nurses and GPs is a prime example of different professions working together with differing values, roles, information needs, and schedules. These differences can lead to tensions and conflicts [14]. Gaining insight into nurses’ perspectives can help to better understand the demands and needs of collaboration, potentially improving care. However, there is a limited body of research on the perspectives of GPs and nursing home staff regarding their collaborative efforts [15–17]. Since effective collaboration is essential for providing quality care in nursing homes, it is important to recognize and understand these tensions [18]. This will enable stakeholders to address them constructively and harness the potential of these tensions to drive positive change within the healthcare system [19]. Digital communication tools present a promising solution to some of these tensions by facilitating quicker, more accurate, and accessible information exchange [20]. Understanding the perspectives of nursing home staff on these tools is crucial in developing solutions that meet their specific needs. However, the current literature does not sufficiently address these perspectives.

## 2. Theoretical background

### 2.1. Paradox theory

The interprofessional collaboration in the setting of nursing homes grows increasingly complex through demographic change and a shortage of healthcare workers, as outlined above. As neither the complexity of care nor the tensions themselves are likely to disappear within the health sector, a better understanding is necessary to allow an approach through which tension can encourage solutions and synergies [14]. Researchers need to understand why tensions emerge, how to categorise them, and finally, how to manage them successfully into positive activators for change [21]. How to cope with conflicting demands and how to use the possible potential of tensions have long been research subjects in management studies. As healthcare systems become increasingly complex and intertwined, Haring and Gersch emphasize the importance of management study approaches, such as paradox theory, for healthcare studies [22]. However, researchers need to adapt these theories to suit the healthcare system and not use them indiscriminately as a template [23].

In the following, we present and discuss paradox theory as the theoretical background for our data and adapt it to improve its fit with healthcare. Applying this framework enables a nuanced understanding of the complex dynamics between nursing homes and GPs. We will follow Haring’s line of reasoning to do so [21].

### 2.2. Organisational tensions

Paradox studies approach organisational tensions in complex systems to explore ‘how organizations can attend to competing demands simultaneously’ [14]. Smith and Lewis define a paradox ‘as contradictory yet interrelated elements that exist simultaneously and persist over time’ and refer to organisational tensions as conflicts arising from opposing, sometimes conflicting views and demands within an organisational system [14]. Thus, organisational tensions, categorised as organising, performing, belonging, and learning, can either hinder or drive innovation. In paradox theory literature, this potential is referred to as virtuous or vicious cycles [24]. An example of a vicious cycle within our setting would be a conflict between a nurse and a GP disrupting the collaboration. Their impaired collaboration then heightens the conflict and causes further disruption. Conversely, a virtuous cycle can be reached by a tension leading to progress within the collaboration, improving the interprofessional work, and resulting in a better quality of care. An example could be a similar conflict leading to the implementation of an innovation that enhances the collaboration. The aim in analysing tensions is to harness their potential to promote virtuous cycles as explained above, whilst reducing them where they cause vicious cycles. This underlines the need for understanding different tensions to develop coping strategies. As Voorhees et al. stated, paradox theory ‘represents an effort to shape the complexity of interrelated problems into something that can be addressed, which is rooted in a strong theoretical foundation’ [25].

### 2.3. Solution strategies

Smith and Lewis describe three solution strategies when approaching organisational tensions that can develop tensions into virtuous cycles [14]. First, ‘either-or’ approaches separate the tensions temporally or spatially. Second, ‘both-and’ approaches try to bring tensions into an equilibrium. Finally, ‘more-than’ approaches seek to develop a synergy that accommodates both poles and creates something new, often through dialectical reasoning and ongoing adaptation and reflexivity.

## 3. Study

### 3.1. Aim

Our aim was to investigate how nursing home staff view communication with GPs. Focusing on current communication issues and communication channels, as well as possible digital communication options, we wanted to explore nurses’ perspectives on digital options as solutions to current problems. Our study specifically examined attitudes toward digital solutions without further exploring other potential strategies for improving the quality of medical care in nursing homes.

## 4. Methods

### 4.1. Study design

To explore the complex relationships and interdependencies between nurses and GPs, as well as nurses’ perspectives on communication pathways, we employed a qualitative methodology using guided interviews. We developed this study’s topic guide from a quantitative GP survey on communication pathways with nursing homes, conducted by some of the authors (EK, KD, AB, SU, TF) to deepen our understanding of nurses’ perspectives [21,26]. Our reporting adheres to the Consolidated Criteria for Reporting Qualitative research (COREQ) [27]. Our checklist is available in the Supplementary Material (see Supplement 1).

The interview guide based on the above-mentioned quantitative GP survey, as well as a first literature research following established procedure was developed by an interprofessional team (KD, EK, AB) [28]. The guide was pilot tested with three laypeople. Only minor changes to the guide were necessary to ensure clarity. This guide was then pilot tested with a geriatric nurse and no more changes were necessary after this last pilot. However, we did not include this pilot interview into the data analysis, as the nurse was based outside the catchment area of our study. Our interview guide contained open questions on communication, communication pathways with GPs, current problems concerning their collaboration with GPs, as well as sociodemographic questions. Lastly, the guide suggested possible digital options for typical communication topics (see Supplement 2). Author KD described a hypothetical shared communication software, which could be used by GPs and nurses alike. We asked participants to share their opinions, rate these, and to add ideas for improvement.

### 4.2. Study setting and recruitment

To recruit study participants, nursing homes in the German regions of rural Saxony-Anhalt, west-central Saxony, and Halle (Saale) were contacted using a database of online contact details, following convenience sampling principles [29]. Hereby, all heads of nursing homes in this sample were contacted *via* phone and fax, or digitally, if an email address was available. Nurses, heads of resident care units, heads of nursing, and nursing home directors were included to ensure diverse perspectives, with further contacts obtained *via* snowball sampling. When participants agreed to participate, we tried to generate a heterogenic sample in terms of age, gender, and work experience through purposive sampling [30]. We gave participants a short overview of the aim of our study and introduced KD as a medical student in pursuit of her doctoral degree, who conducted all interviews. No other relationship with the participants was established. Recruitment occurred from January 2021 to April 2022, but faced challenges due to the COVID-19 pandemic.: To maintain consistency in the sample’s context recruitment concluded when pandemic containment measures ended.

### 4.3. Inclusion and exclusion criteria

Participants were nursing home employees in Saxony-Anhalt and Saxony of all genders, who were over the age of eighteen and spoke German.

### 4.4. Data collection

We included 13 interviews in our study. No participant dropped out after agreeing to participate and we did not repeat interviews. 10 interviews were conducted *via* telephone and 3 face-to-face. We offered digital options to interview each participant, but all declined. All interviewees were in their place of work and had a personal room or office to conduct the interview alone. However, the first 2 participants were interviewed together, as the participants had requested this. Interviews were audio recorded; in addition, KD made field notes during and after the interviews. The interviews lasted between 20 and 41 min, with an average length of 34 min. KD transcribed the interviews manually and verbatim using the F4 transcript software. The accuracy of each transcript was validated against the original recordings. After transcript validation, we deleted the recordings to ensure participants’ anonymity. We did not return interviews nor ask participants for feedback on the findings due to their limited time. A sociodemographic overview of participants is included in Table 1 in our results section.

### 4.5. Data analysis

We analysed the data thematically [31], coding and sorting the text material from the transcribed interviews using MAXQDA software (version 2024) into our coding system. Main themes were constructed deductively based on the research question and the theoretical framework; subthemes were derived inductively from the data. Two coders (KD, JG) coded the first two interviews together, generating an initial coding system. We then separately coded a third interview and discussed the findings. If we were not unanimous, we consulted a third researcher for consensus. This coding system was then applied to all the data by KD and was discussed with JG, EK, and AF throughout the process. KD applied this initial coding system to the remaining data, adding subthemes throughout the process. The altered coding system was once again applied to all of the data, this approach provided a structured understanding of underlying challenges and potential solutions.

To connect this research with broader debates in management and organisation studies, we apply paradox theory as a framing for our findings [32]. Paradox theory’s categories of organisational tensions and solution strategies were defined by Smith and Lewis and further described by Putnam et al. and Gregory et al. [14,24,33,34]. To apply them to our coding system, we define them within the interprofessional work relationship of nursing home staff and GPs. Thus, we will sort mentioned communication problems into paradox theory’s categories of tensions and digital options into the aforementioned solution strategies.

### 4.6. Ethical considerations

Ethical clearance (2021-013) was obtained from Martin Luther University’s ethics committee for the medical faculty. Before each interview, we obtained verbal consent, and for interviews held face to face, we also obtained written consent. Participants were informed that their participation was anonymous. KD substituted names and nursing homes with letter codes, ensuring no direct identifiers remained in the dataset.

### 4.7. Rigour and reflexivity

We tried to ensure rigour through a collaborative coding process and discussing our methodology with other scientists [35]. The project team that conducted this research comprised multiple professions, among them nursing, medicine, psychotherapy, social anthropology, and epidemiology. TF is a General Practitioner. Coding was mostly done by four people. All four are MDs: KD and JG are in pursuit of their doctoral degree, EK is a PhD (all three at the Institute of Family Medicine, in Halle, Germany), and AF is an associate professor. We acknowledge that the introduction of KD as a then medical student in pursuit of her doctoral degree may have caused a potential bias in participants and may have influenced their responses in a way that their critique of medical doctors might have been attenuated due to social desirability. We discussed our approach and methodology several times in a qualitative methods workshop with an even more multidisciplinary group comprising colleagues from, among others, rehabilitation sciences, nursing, language therapy. These multiple exchanges within and beyond the group addressed potential biases in our approach, such as researcher confirmation bias and interviewer observation bias and allowed for better reflection by the researchers conducting the analysis, as the material was viewed from multiple perspectives. We tried to address researcher confirmation bias through a clear audit trail, triangulation, and the aforementioned peer debriefing. We addressed interviewer observation bias through our structured interview guide, recording, and transcription. The coding system was kept adaptable, which allowed us to respond to this in-depth examination of the material. In the exchange, we were also able to establish that our coding system was reasonable and that other qualitative researchers would have identified similar categories. During a second review of the material by KD, the coding system and thus the analysis proved to be stable.

## 5. Results

We interviewed 13 participants from 7 different nursing homes (8 women and 5 men). Their ages ranged from 30 to 56 and their work experience varied between 4 and 34 years. The nursing homes cooperated with 4 to over 15 GPs. Please refer to Table 1 for further information.

**Figure 1.**
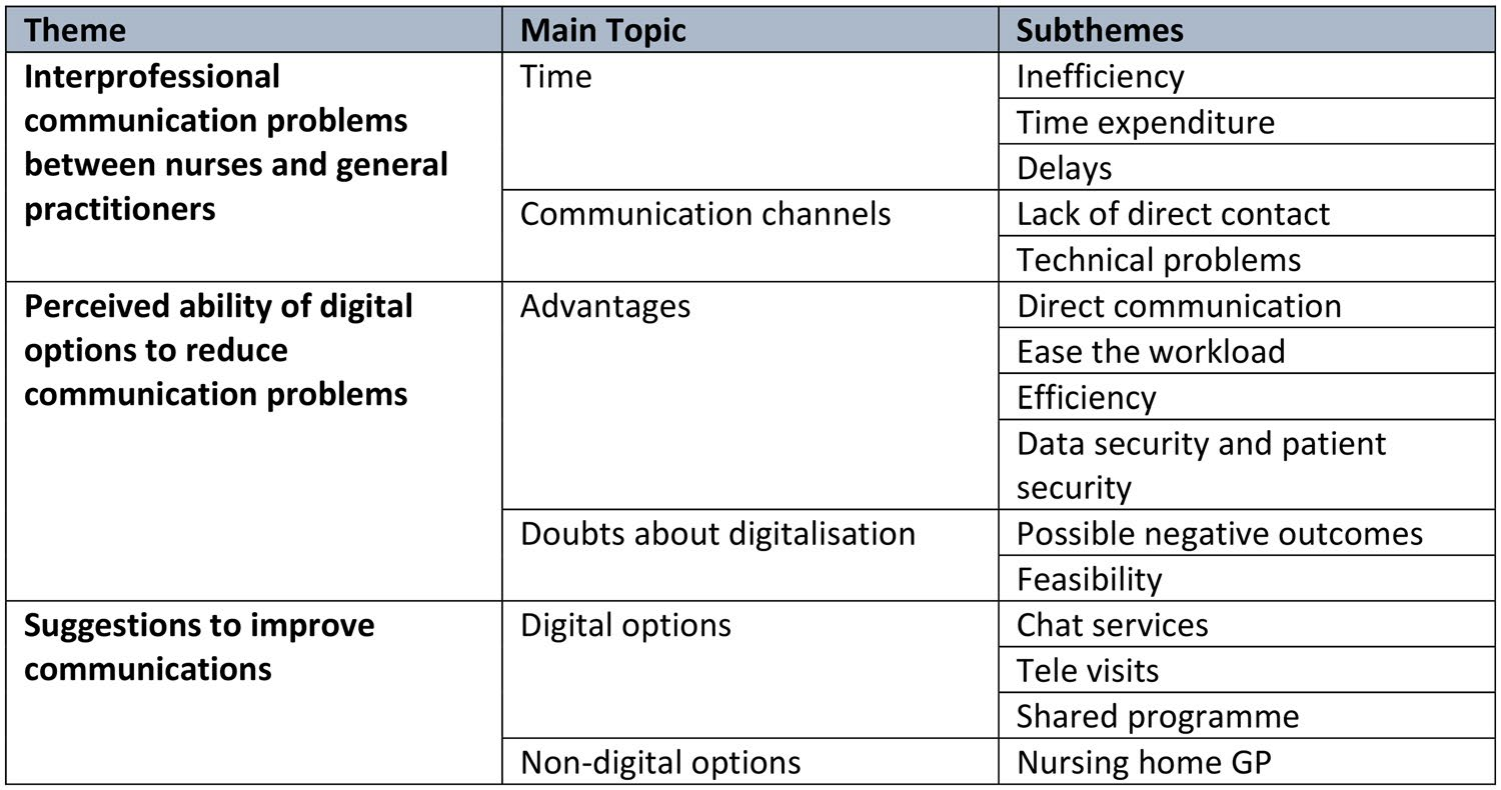
Theme map.

To structure our results, we organized them into three main themes: interprofessional challenges with GPs, perceived potential of digital options to mitigate these interprofessional challenges and participants’ suggestions to improve communications. The results are visualized in a theme map (Figure 1) at the end of this chapter.

### 5.1. Interprofessional communication problems between nurses and general practitioners

Interestingly, even participants who described the cooperation as excellent identified several issues. Time was mentioned as a central concern, with participants describing their interactions with GPs as inefficient, time-intensive, and frequently delayed. Further key problems were difficulty reaching GPs directly and technical challenges with communication channels like fax or phone.

#### 5.1.1. Time

##### 5.1.1.1. Inefficiency

Inefficiency was a dominating aspect of respondent’s narration about problems when working with GPs and was perceived to lead to an avoidable expenditure of time, a critical, and often limited, resource.

*“I need to record the doctor’s ward round, and the doctor has to record his rounds as well accordingly. I believe that to tie up capacities unnecessarily.” -4ED*

The limited availability of communication channels beyond phone and fax also hindered effective record sharing. This inefficiency was reported to create additional workload, often stemming from gaps in communication or the unavailability of the GP. Thus, it was one of the main reasons for the wish to communicate more digitally.

##### 5.1.1.2. Time expenditure

Time cost was mentioned as a problem by interviewees, as they felt to not have enough time with patients. Particularly, nurses felt that infrequent visits by GPs could not be adequately replaced by existing communication pathways, which often led to increased time demands on their part.

*“That’s actually very aggravating for us, the entire situation, when nobody visits to have a look at folks (…), yeah. Actually, it really is a lot with phoning, fax and driving there, collecting prescriptions (…) so you really have a lot to do on top.” −10RR*

##### 5.1.1.3. Delays

Delays, particularly in GPs’ response times, were identified as a significant challenge in collaboration. Some attributed these delays to the heavy workload in GPs’ practices, which led to differing priorities between GPs and nursing homes. Yet participants emphasized that such delays posed major communication issues and increased the workload for nurses. Importantly, delays often lead to avoidable emergency situations and hospital admissions for residents, when nurses evaluated situations as life-threatening after waiting on GPs responses.

*“B: (…) that starting with my request that I sent at 8 o’clock in the morning, 24 hours will pass, before I receive an answer. Of course I will react in the meantime, if the GP doesn’t answer, then I’ll call the on-call-doctor or an emergency doctor, if it’s life-threatening. Yeah, we do that. But for me it really is the reaction time to my request.” −3PH*

#### 5.1.2. Communication channels

##### 5.1.2.1. Lack of direct contact

Nurses expressed significant frustration because they felt unable to contact GPs directly through standard communication methods:

*“Often this ends badly for the patient’s care, because they [GPs] are often not reachable via phone or fax and they answer their mails quite late. Such things happen on a daily basis and you get the feeling that they don’t really care about the patients, maybe because they are not very useful to them, right?” −6WG*

Or – even when communication pathways worked – interviewees felt that the issues sometimes lay in the GPs cabinet, as GP nurses would not forward messages or forget an important aspect.

##### 5.1.2.2. Technical issues

Lastly, participants criticised that technical options that could ease the workload were still not used. This was especially frustrating during the COVID-19 pandemic, as other options were often lacking and faster communication pathways that could fill the gap were not implemented well enough.

*“Well, I believe that, especially in the time of electronic data processing/technology, a faster solution is needed for everybody. I always come back around to the point of saying that we need to use what we have and we are not doing that. Say, all the technology that is just lying around. That’s the case in our nursing home, like if I start to think off all the PCs just lying around, so many laptops. Yes, of course, we use it, but we are not using it ideally and it’s the same in the doctor’s offices.” −3PH*

Participants were frustrated by technical aspects contributing to further time constraints or an unnecessary workload:

*“Currently it’s like, when I find something out, I document it and have to fax here and there 10 times and so you could save all these extra steps, that are in-between.” −4ED*

### 5.2. Perceived ability of digital options to reduce communication problems

#### 5.2.1. Advantages of digital solutions

Participants displayed a broad acceptance towards digital communication options in healthcare. They stated one would have to keep up with the times or saw a necessity for change.

*“Yeah, like I said – right away! Actually, I would really prefer, if we’d do it that way. Away from the fax machine and so on. Phone, WhatsApp, E-Mail. Yeah, I would be definitely all for it.” – 13CJ*

##### 5.2.1.1. Direct communication

Direct digital communication was mentioned often, but interestingly with different meanings. Some perceived it as faster. Others viewed it as a tool for ongoing oversight, enabling GPs to review nursing home documentation more thoroughly:

*“This continuous review will be much easier digitally. Because if we write something into our charts here, the doctors sometimes don’t even notice it and they don’t question it. If I had all of this [the charts] in a programme, there would be a constant check und they could always consult it and things wouldn’t get lost as quickly.” – 6WG*

Lastly, participants understood the advantage of direct communication as being as close as possible to the GP being in the nursing home. One participant felt it could simulate physical proximity to GPs

##### 5.2.1.2. Ease the workload

The point that digital options could ease the workload was a shared topic throughout the interviews. Several aspects that could lighten the load were mentioned and interviewees often pressed that it should happen quickly.

Communication with GPs was perceived as a stress factor that could be reduced through digital options. Furthermore, participants thought it easier to factor into the workday, to make planning with GPs easier and saw it as time-saver for GPs and nursing homes.

*“You could avoid so much labour that way. And to not have to call constantly and that the doctor doesn’t have to come here all the time.” – 8WH*

##### 5.2.1.3. Efficiency

The line between a lightened workload and improved efficiency was often blurry as both could cause each other. We tried to only code statements as ‘Efficient’, when participants mentioned how they could save time. Participants expressed that digital options could make their everyday work more efficient, by reducing time and effort, compared to current options.

*“I wouldn’t have to print the fax, I wouldn’t have to go to the fax machine, I wouldn’t have to wait on the fax receipt and I wouldn’t have to put away the fax receipt.” – 3PH*

Especially, using the computer to contact the GP was seen as logical as both sides had the relevant resident data on their computers anyway. Often participants did not only state how they could save time but saw the advantage in having more time for patients or in being able to help residents faster.

##### 5.2.1.4. Data and patient security

Participants saw data transmitted *via* digital channels as more precise, easier to track, and as mentioned above, reliable. Especially in comparison to orders given over the telephone or handwritten notes on a fax, nurses thought that it could even reduce misunderstandings and errors.

*“The thing, that the doctor’s order through this digital channel would actually be binding and not – just scribbled on some scrap of paper, where you don’t even know if a doctor wrote it and why. So, with this in mind, it would definitely be an advantage – also in comparison to an e-mail.” – 2CB*

Interestingly, participants mentioned data security as an advantage of digital communication within a computer programme between GPs and nursing homes, considering data security only as an issue concerning e-mails or WhatsApp and not necessarily for a specialized software. Similarly, their views differed on ‘old’ communication channels. Whilst some saw the fax as the most secure communication, others questioned the data security of a fax machine. We attribute this to different opinions on data security of fax and phones, but many also thought planned digital communication would prevent pathways seen as less secure, e.g. WhatsApp.

#### 5.2.2. Doubts about digitalisation

##### 5.2.2.1. Possible negative outcomes

Though most had doubts on feasibility, only few mentioned actual disadvantages. The one worry stated was that digital options could further limit personal interactions with GPs for residents and staff in nursing homes. However, others thought that interactions between GPs and residents were already low and could only be improved through new communication pathways:

*“It wouldn’t replace the GPs visit, which is mandatory once a quarter. But it’s not like the GPs visit us more often anyhow and because it’s like so time consuming. The GP’s offices are far away (…) and the gain would be great.” – 7WH*

##### 5.2.2.2. Feasibility

Nurses felt that only upstream solutions through legislation or health insurance companies could make larger implementation feasible, as otherwise it would be too dependent on each GPs office and each nursing home.

*“B: Not on a voluntary basis, no. It [a digital communication programme with GPs] would have to be decided by the legislation, so that everyone does the same.” – 11AJ*

Participants had doubts on the feasibility of digital solutions and some voiced disadvantages they see in a more digitalized communication. Interestingly, even when they stated their doubts, they often would dispel them in the next sentence and emphasized their need for digital solutions.

Participants mentioned financial implications of digitalisation as an important aspect, but did not imply that this could hinder feasibility.

Participants were worried that the transition to digital communication could be difficult for some colleagues. However, many nursing homes have already made the change to digital nursing charts within the nursing homes and would draw comparisons between these innovations.

*“B: So, we had/three years ago we started with digital documentation and the first time I saw it, I thought ‘We’ll never get the hang of it! (…) Yeah. And we still discover new things every day and we all say: ‘How did we do it before?’ So, yeah. Starting with readability and everything is much clearer.”– 11AJ*

### 5.3. Suggestions to improve communication

As mentioned in our subtheme Advantages of digital solutions, participants suggested their own ideas for digital options in communication with GPs. Following, we present the 3 most pervasive suggestions for digital options. The third suggestion may have been prompted by the interview guide, as KD described a hypothetical shared communication software, but the aspects and details mentioned below are solely the participants’ ideas.

#### 5.3.1. Digital options

##### 5.3.1.1. Chat services

Nurses wished for WhatsApp or other chat or text services to become a communication pathway with GPs. They viewed it as quicker with a lower threshold, as the GP would check his phone often, and it would allow for asynchronous communications when it best suited both sides.

*„B: It [WhatsApp] would really help us so much with the communication and also with treatment/I really have to say, it would help us so much with treatments. Because the GP is quicker to reach.” – 10CJ*

Despite some concerns about data security, participants acknowledged the practicality of such tools, some also admitted to having had used WhatsApp in the past to get a quicker reply than fax or landline.

##### 5.3.1.2. Tele visits

Nurses mentioned digital visits and ward rounds as important digital options. As we collected our data during the pandemic, medical doctors began offering tele consults, but only one of our participating nursing homes had any experience with them. They viewed it as a time-saver and as more direct as the doctor could assess personally how his patients were doing.

*„B: (…) Well I’d (…) like to change it, so that colleagues can go there [to the residents] with a tablet, that they can skype and that the doctor can watch from his office. That would be such a time-saver for many people, I’d say. And you don’t have to call and call again and the doctor doesn’t need to visit all the time.” – 8WH*

As mentioned above, nurses felt GPs did not visit their nursing homes often and that a digital ward round would improve rather than lessen contact between residents and their GPs as they felt GPs did not visit often in person.

##### 5.3.1.3. Shared health information programme

According to nurses, a shared programme to access patient files would allow the GPs check-up on their patients’ general well-being from their own offices and help them stay in the loop on their patients in nursing homes. That could be beneficial as nurses mentioned most GPs did not visit every week. Furthermore, they viewed it as a great option to allow GPs and nurses to interact with each other and to change or document things in the patients’ file, which the other could respond to.

*„B: It would be easier, in this day and age – especially because we just switched to digital documentation – to link this programme as a form of interface [between the GP and the nursing home], that everything runs via e-mail, but compliant with data protection.” – 3PH*

These suggestions underline the potential of digital solutions to address systemic inefficiencies and enhance interprofessional collaboration.

#### 5.3.2. Non-digital options

##### 5.3.2.1. Nursing home GP

Though not a digital option, an interesting aspect participants mentioned was their wish for a general practitioner who was the responsible GP for all their residents. They envisioned GPs assigned exclusively to nursing homes, improving both communication and medical routines for staff and residents. Others even imagined a broader concept, including specialists for neurology and dentistry:

*“B: It would really be better, to imagine having a nursing home GP, a nursing home neurologist and a nursing home dentist. So if they attended to us regularly, if everyone was in this portal and everything was like this/that is a really nice dream.” – 3 ED*

## 6. Discussion

This study reports on nurses’ perspectives on current communication issues with GPs, and possible digital solutions for these problems. The lack of direct contact, technical issues, and mostly time-related problems such as delays, inefficiency, or time expenditure were participants’ main concerns regarding interprofessional communication. All participants made clear that the current situation would need to change. We believe this need was further heightened within the pandemic context of our data collection. Participants showed a general openness towards digitalisation, regardless of age or work experience. Digital options mentioned by nurses that could improve collaboration with GPs were tele consults, chat services, and a shared digital programme. They viewed digital communication pathways as a necessity. Participants hoped digital options could lessen the workload, reduce misunderstanding, and be more efficient, direct, and secure.

### 6.1. Interprofessional communication problems as organisational tensions

The issues highlighted by participants often reflect underlying tensions [36]. *Organising tensions* occur when collaborating systems, such as nursing homes and GPs, have differing demands and processes to reach their objectives. As the two collaborating systems have different processes, extra time is needed to ensure that both systems have the same information, resulting in inefficient use of time [37]. For example, the lack of a shared communication system between GPs and nursing homes leads to information loss or redundant documentation. In addition, delays in the communication of information between the two systems and the lack of direct contact often arose from similar situations and can also be sorted into *organising tensions*. Nurses were frustrated that they could not reach the GP directly and promptly, and felt that this caused further delays.

However, often participants felt as if problems were not only an organising issue but rather the result of different priorities between the nursing home and the GP’s cabinet. These could be understood as either *performing tensions*, which result from different goals set by different stakeholders, or *belonging tensions* that are more intrapersonal, with different identities of an individual either clashing with each other or with roles defined by the collaboration. If goals between GPs and nurses diverged, we defined these tensions as *performing tensions*. Often, participants mentioned that they could not reach the GP during their office hours, and assumed that the GP prioritized the patients in their cabinet over their nursing home patients. Some participants went so far as to state that they felt like the GPs did not care about their nursing home residents. This seemed as a more intrapersonal conflict, where the GP’s multiple roles clashed with each other. These clashes can be seen as examples of *belonging tensions*. Jarzabkowski et al. concluded that *belonging*, *performing*, and *organising tensions* often co-evolve leading to role conflict [36]. Furthermore, the coexistence of an intrapersonal and a role conflict emphasises one of the difficulties in applying paradox theory to this specific collaboration. We found it almost impossible to separate *belonging and performing tensions* when the GPs were concerned. GPs are individuals with many differing roles. As GPs in Germany often have their own doctor’s office [38], they are medical doctors as well as entrepreneurs [39]. Additionally, their goal to take care of their patients is already conflicting as the two patient groups in their office and in nursing homes take time and resources away from each other. Thus, to describe these two tensions separately is almost impossible. We could use cross-cutting tensions described by Smith and Lewis [14], but this would fall short here. It is not a conflict of *performing tensions* with *belonging tensions*, but rather a conflict of *performing and belonging tensions* intertwined. Putnam et al. define this phenomenon as paradox knotting [33].

*Learning tensions* appear when innovation changes dynamic systems. Thus, *learning tensions* were mostly mentioned when participants talked about digital possibilities. The implementation of using computers in nursing homes and the electronic patient file were mentioned and are good examples of *learning tensions* [40]. Interestingly, most participants used them as an encouragement for further digital communication, even though they did describe difficulties for nursing home staff in adapting to them. But because most participants had overcome this hurdle and advantages of this new system had become clear, they saw the difficulties not as unsurmountable, but rather as a normal process of innovation [41].

These descriptions of learning tensions encompass the potential of tensions well. For one, they act as a driver for innovation due to a drive to cope with that tension. Nurses voice their frustration over interprofessional communication problems and are open for more digital communication because of it. But the second potential of tensions mentioned in our interviews is even more interesting: Participants used learning tensions as an encouragement for further innovation, because they were able to cope with them. This shows how important coping strategies are to harness the potential of tensions [42]. To summarize, tensions demand innovation in form of coping strategies, if they are not dealt with. But they can also be an accelerator for innovation, especially when they are well managed, as they have the potential to reduce fear of innovation and change [41].

### 6.2. Digital options as solution strategies

Smith and Lewis describe three solution strategies when approaching organisational tensions that can develop tensions into virtuous cycles [14]. ‘Either/Or’ Solutions separate the different poles of paradox to reduce tension, ‘Both/And’ solutions try to balance tensions, and ‘More/Than’ Solutions try to develop something new that encompasses both poles of a paradox to use these tensions synergistically. Thus, we sorted participants’ suggestions into these three categories.

#### 6.2.1. Either/or

These approaches separate the tensions temporally or spatially. An example were chat services, as they were seen as easy and quick communication pathways by participants. As most communication channels they allow a physical separation, but the asynchronous communication through texts or voice messages enables a temporal separation as well. They argued that both they and the GP have their mobile phone on them all the time anyway, and it would be easier to send a short message or a photograph or to type a quick reply in a short break. However, often they felt that chat services such as WhatsApp were forbidden to use, either by their institution or because they themselves saw it as a data breach towards residents [43]. These participants wished more precisely for a data protection compliant communication system.

#### 6.2.2. Both/and

These approaches try to bring tensions into an equilibrium. Tele consults are already implemented in some countries to provide GP care to residents of nursing homes [44]. However, in Germany, they are still rare [45]. In our study, only one nursing home had used tele consults with a GP. Nevertheless, many of our participants provided this solution as an example of further digital communication with GPs that they wished for. From their perspective, tele consults can reduce several of the tensions mentioned as they would be quicker and reduce mistakes. Interestingly, our participants were generally not worried that a tele consult could reduce personal contact with GPs as they told us that many GPs did not visit more often than the mandatory quarterly visit and felt that it could further contact with the GP.

We view tele consults or digital ward rounds as an example for a ‘*both/and’* solution strategy. Patients and nurses have more personal contact with the GP, whilst still being more efficient and time saving for all health workers involved. This balance between the two tensions is a typical aspect of *‘both/and’* solutions [14]. However, the aspect of separating two poles of tensions spatially is normally an aspect of an *‘either/or’* response. As digital options allow remote yet personal, direct communication, these solution strategies of *‘either/or’* and *‘both/and’* merge [46].

#### 6.2.3. More/than

‘More/Than’ approaches seek to develop a synergy that accommodates both poles and create something new. The issues of the German healthcare system in general and problems between GPs and nursing homes specifically have created the need for a shared digital programme in our participants. This could not only solve some of their problems with GPs but improve collaboration between GPs and nursing homes altogether. Thus, a shared digital programme as a ‘*more/than’* strategy ‘[creates] a new element that surpasses the existing divergences’ [21]. Participants saw it as efficient time-saver without delays. Moreover, it can enable direct contact and save resources. A shared digital programme offers a similar solution to the dilemma between taking care of nursing homes residents and additional travel time for GPs as digital consults, but with the added improvement of asynchronous communication. Additionally, nurses mentioned a reduced error rate for digital documentation, this is supported by the findings of Albagmi et al.’s systematic review of electronic medical records [47].

Even though it is not an example of a digital solution, the ‘nursing home GP’ participants envisioned, meaning a GP assigned exclusively to nursing homes or a GP responsible for all residents of a nursing home is an excellent example of a More/Than approach. As mentioned above it would be ‘a new element’ to overcome current issues, as it would lead to more direct contact, would reduce technical issues, and temporal issues such as delays.

### 6.3. Strength and limitations

Our study explored perspectives of a diverse group of nursing home staff in Germany and thus can improve the practical relevance of digital solutions in healthcare, as engaging possible users improves understanding and implementation of innovations [48]. We addressed the four defining aspects of qualitative research proposed by Aspers and Corte by maintaining *closeness* through in-depth interviews in nursing homes, which we transcribed verbatim following a *process*-oriented, iterative approach [49]. Through our qualitative methodology and peer debriefing, we were able to *understand* which problems nursing home staff encounter in collaboration with GPs. We ensured *distinction* by using paradox theory to divide communication issues into different categories of tensions, which again improved *understanding*. In addition, the dialogue between paradox theory and our data furthers the cross-linking of this theory and health studies. Paradox theory can help us to see contradictions in health system operations and structures, which can offer new potential approaches to better the quality of collaboration between nursing homes and GPs and could improve the quality of care for residents.

A background in nursing may have led the researcher to focus on other topics, or alternatively, there may be a greater understanding of the topics due to personal experience in this area. None of the authors have experience working as nursing home staff, which can create researcher bias or a risk of missing perspectives. In chapter 4.7, we explained how we attempted to address researcher confirmation bias and interviewer observation bias; however, these must, of course, be acknowledged as potential limitations.

We tried to ensure the trustworthiness of our results, as formalized by Lincoln and Guba in 1985 [50]. To enhance credibility (internal validity), we employed multiple perspectives and peer debriefing in method workshops and reflexivity through a field note journal. Resource and time limitations (mostly on the side of our respondents) prevented member checking, which could have enhanced credibility further. We maintained an audit trail throughout the research process and provided detailed participant demographics and described the context of our study. However, the role of the GP differs in different countries and issues between nursing home staff and GPs may vary internationally. Thus, our findings are not generalizable. Due to pandemic regulations, only three interviews were conducted in person, all other *via* the phone, this reduced non-verbal communication [51]. Furthermore, the pandemic may have hindered recruitment as nursing homes were hit especially hard and often were overburdened with work [52]. Yet the pandemic context might have promoted interest in our study. We considered information power, following Malterud et al. in assessing the adequacy of our data [53]. Our 13 participants were recruited as explained above and we used paradox theory as theoretical guidance, improving the information power of our study, and with the resulting material, we were able to sufficiently respond to the research question. As participants were informed of the topic of our interviews as communication with GPs and possible digital options, this may have influenced their views on digitalisation and may have introduced selection bias. Future research should take nursing home staff′s perspectives more into account and test implementation of digital options between nursing homes and GPs. We recommend the further use of paradox theory to understand complex situations with the healthcare system and have given an application example in this study.

## 7. Conclusion

Our study showed that nurses view digital solutions as valid for current problems in the collaboration with GPs. This work focused on attitudes toward digital solutions, not other potential strategies for improving the quality of care in nursing homes. Our study is an example of understanding current problems of participants, such as delays, inefficiency, technical issues, or lack of direct contact, and their underlying tensions to develop fitting solution strategies that participants want. The dialogue between our data and paradox theory furthers a better understanding of tensions in healthcare collaboration and possible solutions strategies. We hope our results can enable the development of practical, sustainable solutions to improve care delivery in nursing homes, as there is a need for guidelines and care strategies for this particularly vulnerable patient group.

## 8. Clinical relevance

Our research furthers the cross-linking of paradox theory and health studies. As mentioned above, this could improve the quality of collaboration between nursing homes and GPs and could improve the quality of care for residents. As stakeholders wish for digital options, upstream solutions need to be implemented to improve collaboration between GPs and nursing homes and to improve residents’ care. Digital solutions are not intended to resolve systemic issues but to help mitigate their impact and enhance care delivery under current conditions. Thus, our findings indicate that digitalization represents a valuable support strategy within existing constraints.

## Supplementary Material

Interview Guide English.docx

COREQ_Checklist_Revised.pdf

## Data Availability

The data that support the findings of this study are available on request from the corresponding author, KD. The data are not publicly available, as the participants’ anonymity and privacy may be at risk if all collected qualitative data is shared.
