# Supplementary material for: ‘The GP’s offices are far away and the gain would be great.’—A qualitative study on nursing home staff’s perspectives on digital solutions for communication issues with general practitioners
Source: Scand J Prim Health Care. 2026 Apr 6;44(1):2638515. doi: 10.1080/02813432.2026.2638515 (PMC13055025; doi:10.1080/02813432.2026.2638515)
Supplement: Interview Guide English.docx [file IPRI_A_2638515_SM9019.docx]

**Current interview guide for interviews „chances and hurdles in the communication of nursing homes and General practioners“ (working title)**

| **Interview - Problems and chances in the communication of nursing homes and general practitioners - Perspectives of nursing home employees** | |
| --- | --- |
| Interviewer: | Date: |
| Nursing home / employee: | |
| - Introduction (name, institution) - Explanation of the objectives of the interviews (perspectives/attitudes, including limitations, problems, criteria, opportunities, and potential solutions) - "Emphasising the importance of collaboration between nursing homes and general practitioners" - Ask permission for audio recordings - Emphasize the anonymity of the interviews (neither nursing homes nor individual staff members can be identified) | |

**Start the recording!**

| **Statement/ Question type** | **possible phrasing / follow-up questions** |
| --- | --- |
| **Greeting** | Good day, and welcome to today’s interview on the topic of communication and collaboration with general practitioners. |
| **Explain the aim of our study** | The aim of this interview ist o explore how primary care for patients living in nursing homes can be improved and simplified through digital options. |
| **Ask permission again (on the record)** | Do you agree to have this interview audio-recorded for documentation purposes? Of course, this survey is anonymous, and it will not be possible to identify the nursing home or any individual staff member from the recordings during later analysis. |

**Sociodemographic questions**

1. How many years have you worked in nursing homes?
2. What is your age?
3. What is your formal qualification/title?
4. What is your area of work or role, and since when?
5. How many GPs does your nursing home collaborate with?

**Questions relating to communication with GPs**

1. **What role does communication with general practitioners play in your daily work?**
   1. How would you rate communication with GPs on a scale from 1 (terrible) to 10 (excellent)?
   2. Could you explain your rating?
2. Which communication channels do you use with GPs
   1. What are the advantages of each communication method? (Phone, fax, post, email)
   2. Has communication changed in recent years
3. What are the main problems in communicating with GPs?
   1. Please give some examples.
4. Do you currently see a need to change your communication with GPs?
   1. If so, what specifically?
   2. What is your opinion on more digital communication with general practitioners?

**Use cases**

Explain each use case, then ask the following questions.

1. Vital signs
2. Medication plan changes
3. Adjustment of insulin regimen
4. Wound documentation
5. Follow-up prescription

Follow-up questions:

1. Do you have any questions?
2. Could you please summarize the use case?
3. Could this use case be an improvement for your daily work? Or: How would you rate this digital proposal?
4. Do you have any additional comments on this use case?

After the use cases

1. Do you have any ideas for additional digital use cases?
2. What is your opinion on more digital communication with general practitioners after our interviews?
3. Is there anything you would like to add that we haven’t discussed yet?

**Announce the end of the interview and stop the recording! Thank the interview partner.**
